# Annotations

**Published:** 1910-02-12

**Authors:** 


### Annotations.

### Deaths under Anæsthetics.

A summary of anæsthetics and operations for the year 1909 has been published by the authorities of Guy's Hospital in last week's issue of the *Hospital Gazette*, together with an appendix giving brief histories of the cases in which patients have died "under an anæsthetic." This appendix, of course, enumerates only cases in which patients have died while actually on the operating table before or during the operation. We have already pointed out that the term "death under an anæsthetic" is open to widely different interpretations, and that, as a matter of fact, it is interpreted in widely various ways by different reporters. For statistical purposes, however, the limitation of the term to such cases as are given in this appendix is to be commended, although it is worth while to point out that in three of the five cases of which details are published it is an open question whether or not the anæsthetic was the main cause of the death of the patient. It would be an excellent thing if other hospitals followed this example and published short notes of their fatalities under anæsthetics. At present it is difficult to get details of such cases except by collating the coroners' reports—a method which is open to many objections—and it is only fair that the staff anæsthetists should give their versions of the cases.

### Co-education and Deterioration.

In a recent book on America, Mr. Alexander Francis runs a tilt against the co-education of boys and girls, and the employment of women teachers in boys' classes. He frames a terrible indictment against American boyhood. According to him the boy in the States is becoming an inferior copy of the girl, winning a girl's gentleness and sensitiveness, but fast losing the splendid virility of the early settlers. To-day there are 109,179 male and no fewer than 356,884 female teachers in America, or, as he expresses it, "throughout the country, of every four teachers three are women." "In the high schools boys of 18 years of age, whose physical nature needs the most careful development, are taught by women who sometimes are not many years their seniors. Men have told me that they now recognise that serious injury was wrought upon them at that period of their school life when, lonely, shy, and sullen, they were left to fight through their crisis, not knowing that it was a crisis that came to all and was necessary in the development of life." All this is very lachrymose, and doubtless has a grain of truth in it; but educationists in England, not even to speak of their American colleagues, will not accept the indictment in its entirety. The bad effects of co-education should be looked for in the national statistics. No touring journalist, however observant, is capable of judging its influence as a factor in national deterioration, unless he is at the same time a medico-psychiatrist who has made a special study of environmental influences; and no one who has made such a study will for a moment agree that co-education is a serious factor, or even an appreciable factor, in altering the virility of a race. The birth statistics, and those of marriage, flatly contradict such an assumption, so far as America is concerned, and it is the height of absurdity to suggest that the men who fought in the Philippines, who reorganised Cuba, and who are proving themselves the best agriculturists in the world, are less splendidly virile than the early settlers who colonised Pennsylvania. There are undoubtedly factors at work which make for racial deterioration in the United States more potently than, in our opinion, they do in this country; but co-education of the sexes is not one of them. The arguments which are advanced against this system are sentimental, not scientific, and so long as questions in which problems of sex and psychiatry are involved remain in the unsettled condition in which they are at present, it would be absurd to pretend that any discussion on such subjects can be thoroughly scientific. We entirely lack the necessary data on which to base conclusions. To enable us to get such data demands the careful and accurate compilation of a mass of statistics, a close and prolonged study of eugenics, and a systematic comparison of such matters as crime, insanity, mortality, and general signs of progress or retrograde movements, between America and England or Germany. Such a study as this compilation involves will be exceedingly interesting; its results, provided care is taken and fact is not allowed to be overpowered by fancy, are bound to be valuable and instructive. Meanwhile academic disquisitions upon the dangers of co-education and feminine influence upon the manhood and boyhood of the States are as unfruitful and foolish as the ancient scholastic speculations upon the intentions of a hydra in a vacuum, or the corybantic inclinations of death angels on the point of a sewing needle.

### "Truth's" Caution List.

The new edition of *Truth's* Caution List is a publication that should be in the hands of every general practitioner, and allowed a place on the table in every consulting room. Very often the family practitioner is called upon not only to give clinical advice, but also to join in the household council, informally perhaps, but none the less seriously, when matters of general importance are discussed. This is especially the case with poor patients, who often request advice on subjects altogether outside the legitimate scope of the faculty. Sometimes these matters touch very closely upon such subjects as are dealt with in this admirable compilation that *Truth* has so courageously put before the public, and we venture to think that it will greatly add to the general knowledge of the practitioner to be thoroughly familiar with the contents of this Caution List. In it are exposed the wiles of many quacks and experts in the art of practising that deception which the public loves. The quack list alone is worth the shilling which the booklet costs, and it is only one of a dozen other and equally important lists. We can cordially commend the new edition to the attention not only of doctors, but of every layman as well.

